# An Advanced Comprehensive Physiotherapy Management for Empyema Thoracis and Scoliosis: A Case Report

**DOI:** 10.7759/cureus.30500

**Published:** 2022-10-20

**Authors:** Anushka Raipure, Ruhi Kumbhare, Rashmi R Walke

**Affiliations:** 1 Cardiorespiratory Physiotherapy, Ravi Nair Physiotherapy College, Datta Meghe Institute of Medical Sciences, Wardha, IND

**Keywords:** tuberculous empyema, scoliosis, pulmonary rehabilitation, physiotherapy, empyema thoracis

## Abstract

Tuberculous empyema is caused by *Mycobacterium tuberculosis* infection of the pleural cavity, resulting in purulent pleural fluid formation. Tuberculous empyema most commonly develops in patients with tuberculous pleuritis treated with artificial pneumothorax. However, it can also develop in patients with chronic tuberculous pleuritis, usually in patients with pulmonary tuberculosis treated with antituberculous chemotherapy. Scoliosis is a three-dimensional spine deformity caused by several factors, including genetic susceptibility, anterior and posterior spinal development imbalance, and connective tissue abnormalities (skeletal muscle and nerves). Although surgery is the most talked-about treatment option, there is high-quality evidence suggesting the use of conservative therapy in the management of scoliosis. A systematic rehabilitation plan with a variety of approaches was developed, and it was found to be a highly successful protocol for treating the patient’s empyema and scoliosis.

## Introduction

A common pulmonary consequence of tuberculosis in the pre-chemotherapy period was tuberculous empyema. It was either caused by progressive pulmonary tuberculosis or any surgical intervention. Even though its incidence has decreased, it accounts for up to 6% of total empyema cases. In more severe cases of tuberculous, empyema results from cavitation into the pleural space or brutal invasion of the parenchymal focus, contrary to pleural effusion caused by tuberculous pleurisy, wherein hypersensitivity plays an important role [1].

Tuberculous empyema is caused by *Mycobacterium tuberculosis* infection of the pleural cavity, resulting in purulent pleural fluid formation. Tuberculous empyema most commonly develops in patients with tuberculous pleuritis treated with artificial pneumothorax. However, it can also develop in patients with chronic tuberculous pleuritis, usually with trapped lungs, or patients with pulmonary tuberculosis treated with antituberculous chemotherapy [2]. Tuberculous empyema is more common in middle-aged adults. Patients frequently have pulmonary or pleural tuberculosis for more than 10 years before the empyema is discovered [3]. Clinically, symptomatic people with a chronic or subacute clinical picture are characterized by fever, nocturnal sweats, weight loss, and an elevated erythrocyte sedimentation rate. The most common radiological symptom is a pleural-based density. Chest tomography frequently reveals an enlarged rib cage, loculated pleural effusion, and a thick, fibrocalcific pleura. The existence of air-fluid levels verifies the presence of a bronchopleural fistula [4].

Scoliosis roughly affects 3% of the population, mainly females. It is a three-dimensional spine deformity in a three-dimensional plane due to genetics, improper development of the anterior and posterior spine, and connective tissue abnormalities of skeletal muscle and nerves. Surgery is a widely discussed treatment option for scoliosis, but some studies suggest conservative management helps manage scoliosis [5].

## Case presentation

Relatives brought a 35-year-old male, a mason by occupation, with the chief complaints of cough with mucoid expectoration, fever (on and off), low grade and intermittent, loss of appetite, and weight loss for the last two months. He had been experiencing breathlessness for one week. There was no history of hemoptysis or Koch’s contact, and he was an alcoholic for 10 years, 100 mL per day. He stopped consuming alcohol for three months. The patient went to a local hospital two months back. On investigations, a chest X-ray revealed pleural effusion. The physician inserted an intercostal drain (ICD) and drained 2 L of straw-colored fluid. The patient started CAT-I DOTS (Category 1 Directly Observed Therapy). Category 1 treatment was given for the intensive phase, i.e., the first two months. After one month, the patient went for a follow-up when a repeat chest X-ray showed pleural effusion. The physician performed diagnostic tapping and placed an ICD, removing it after draining 200 mL of fluid. Table 1 presents the chronology of the events.

**Table 1:** The timeline of the events. ICD: intercostal drain

Event	Date of event
Date of first ICD insertion and drainage	4/11/21
Date of assessment	31/12/21
Date of second ICD insertion and drainage	2/1/22
Date of commencement of physiotherapy rehabilitation	3/1/22
Date of discharge	8/1/22

On examination, he was conscious, oriented, and afebrile with a pulse rate of 124 beats per minute, blood pressure of 102/70 mmHg, SpO_2_ of 97%, and respiratory rate of 30 breaths per minute. Pallor was present, and jugular venous pressure was normal with no lymphadenopathy and pedal edema. Systemic examination revealed scoliosis and a displaced trachea to the right. Percussion produced a dull sound over the left lower lobe. Dullness was present in (1) the fifth intercostal space in the midclavicular line, (2) the eighth intercostal space in the midaxillary line, and (3) the tenth intercostal space infrascapularly. A succussion splash was present.

Further examination revealed shifting dullness. On auscultation, there was an absent breath sound at the left lower and upper lobes. The left side demonstrated egophony and bronchophony. His X-ray of the cervico-dorsal spine showed kyphosis with scoliosis, minimal cervical spondylosis, left pleural empyema with decreased volume of the left lung with consolidation and fibrosis of the left lower lobe, and pulled mediastinum toward the left. We present a case of left tubercular empyema with mild scoliosis.

Pre-rehabilitation assessment

Table 2 shows the pre-rehabilitation assessment.

**Table 2:** The pre-rehabilitation assessment of the patient. NPRS: Numeric Pain Rating Scale; mMRC: Modified Medical Research Council; WHO-QOL: World Health Organization quality of life

Outcome measures	Results
NPRS	8/10
mMRC grade of syspnea	Grade 2
WHO-QOL	51/100
Six-minute walk test	180 m

Radiological findings

**Figure 1:**
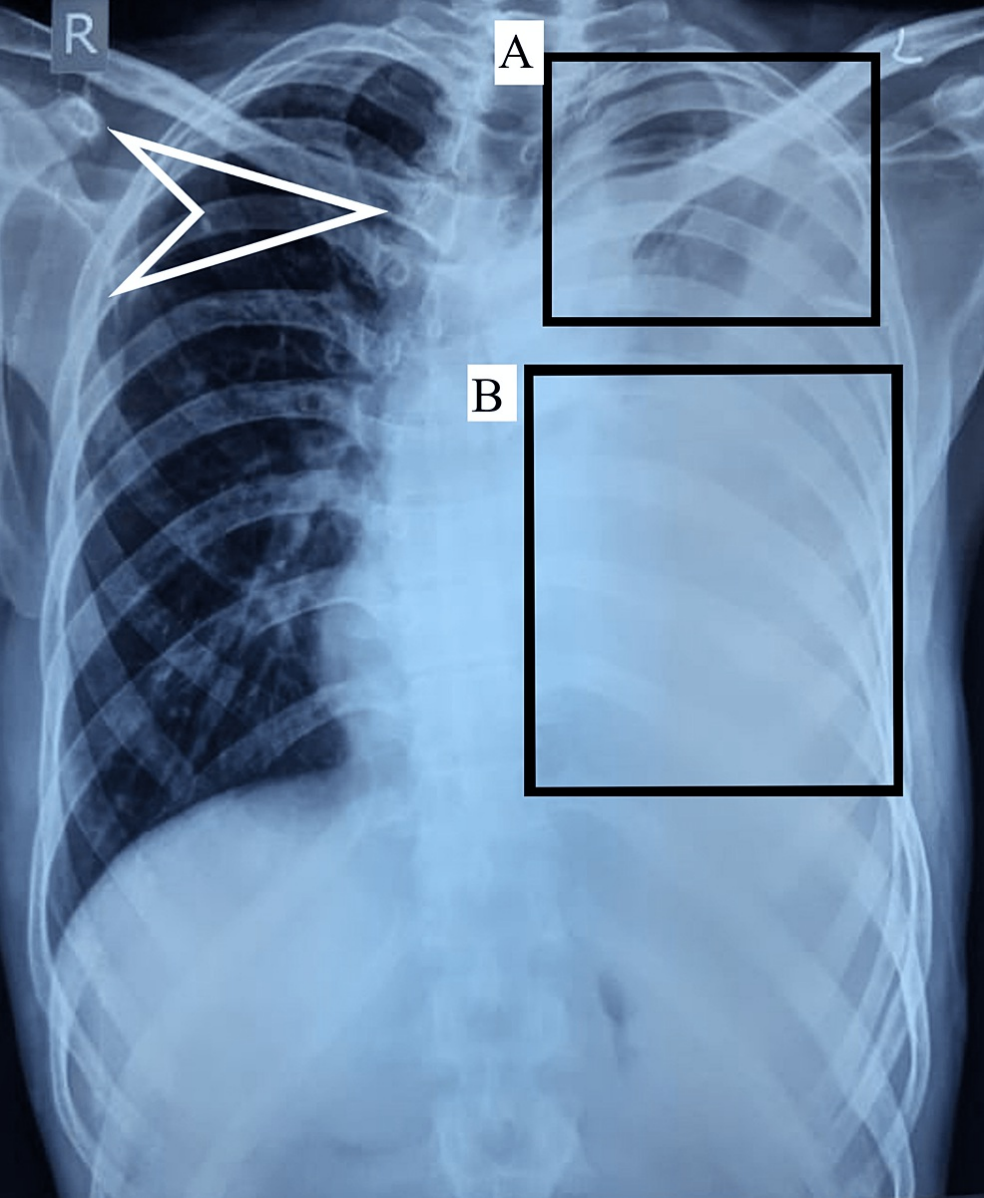
Chest X-ray of the patient taken in the posteroanterior view with haziness seen over the left upper zone (box A) and consolidation in the middle and lower zone (box B) with mild scoliosis (arrow).

**Figure 2:**
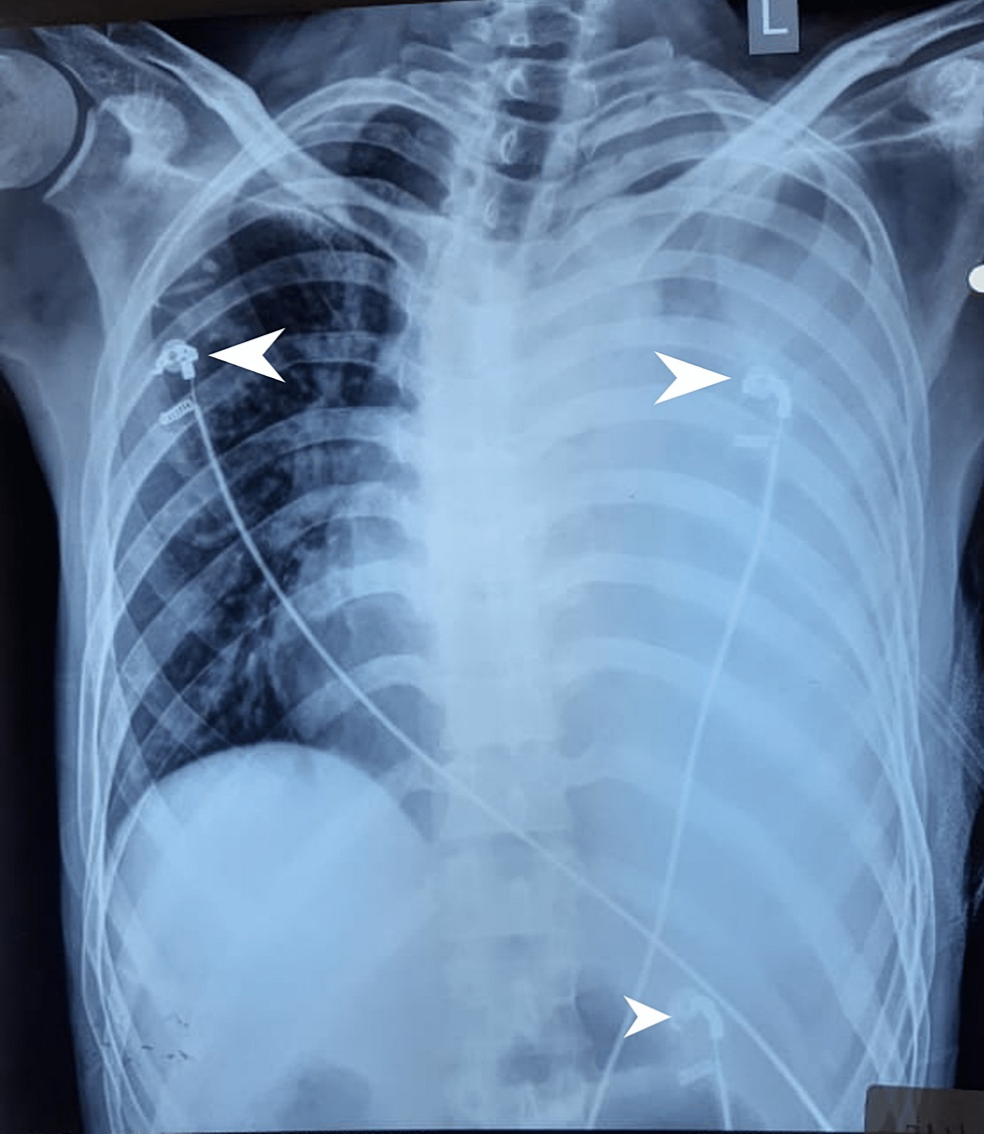
Chest X-ray taken on the posteroanterior view with the intercostal drain in situ.

Figure 1 and Figure 2 show the radiological findings of the patient.

Therapeutic intervention

**Figure 3:**
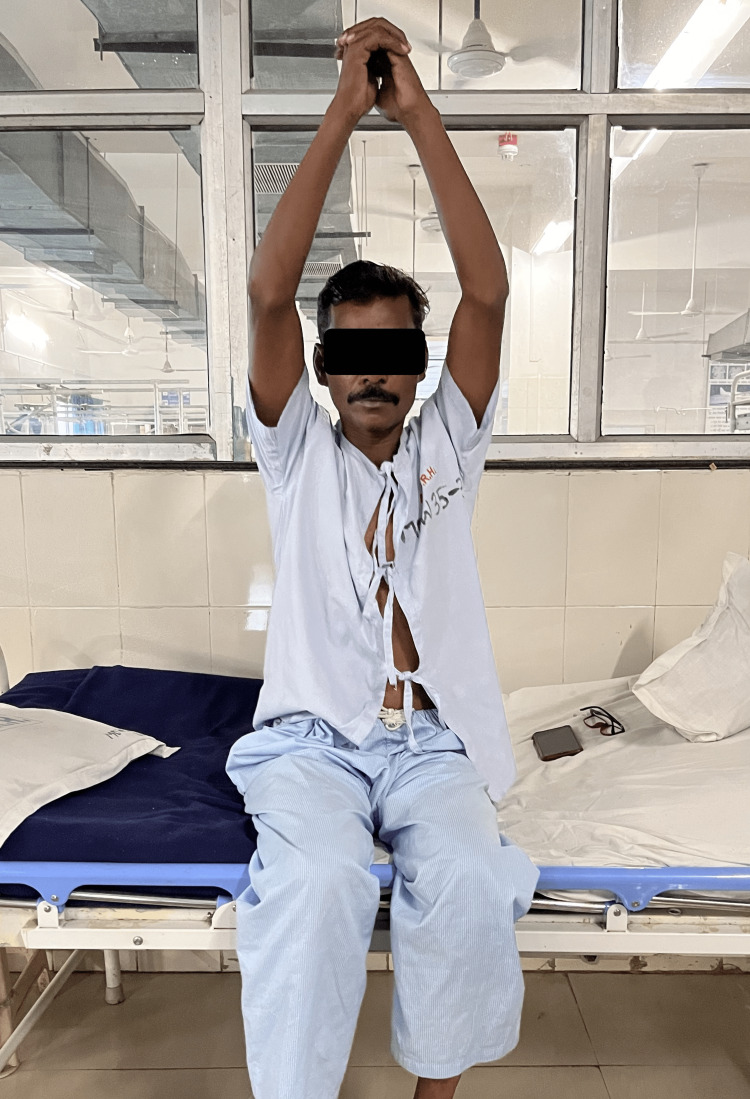
The patient performing thoracic expansion exercises.

Physiotherapy management began as soon as the patient’s ICD tube was removed, with the goal of improving the patient’s ventilation and exercise tolerance capacity, clearing the airways, reducing the effort of breathing, and promoting relaxation. It would enable the patient to carry out daily activities without experiencing weariness or discomfort. The Frequency, Intensity, Time, and Type (FITT) principle guided the design of the physiotherapy interventions. Table 3 shows the treatment administered with goals. Figure 3 shows the patient doing thoracic expansion exercises.

**Table 3:** Physiotherapeutic interventions with goals.

Goals	Rationale	Interventions
Patient education	-	We educated the patient about the disease, its progression, complications, associated illnesses, the importance of physiotherapy, exercise advice, and pain education neuroscience
Diaphragmatic exercises, segmental breathing exercises, and incentive spirometry	For maintaining and regaining total lung volume and capacity	Thrice a day, starting with one set and then progressing to three sets per day till week four
Thoracic expansion exercises	Breathing exercises for minimizing adhesion formation	Twice a day for 10 minutes every day, gradually progressing. Figure 3 shows the patient doing thoracic expansion exercises
Manual chest physiotherapy with positions and active cycle of breathing technique	For clearing lung fields	Manual chest physiotherapy three times per day for up to two weeks. Active cycle of breathing technique twice a day for 10 minutes and progressing gradually
Positioning	The therapist used postural correction to maintain thoracic mobility and healthy posture	The therapist instructed the patient to sit with his shoulder level and weight evenly distributed on both hips
Back strengthening	Increase the maximal number of repetitions	Back strengthening exercises like pelvic bridging, knee-to-chest stretches, and reach-outs in quadruped were initially given for 10 repetitions for one set twice a day and then progressed to 10 repetitions for three sets twice a day till week four
Schroth exercises	To treat scoliosis	Schroth exercises: on the swiss ball in front of a mirror, in the prone position, pelvic tilts, cat and camel, double-leg abdominal press, single-leg balance for 10 repetitions one set twice a day and progressed to 10 repetitions three sets twice a day
Change in position	To avoid bedsores	Frequent changing of position, preferably three hourly, was advised
Upper and lower limb active exercises	To improve mobility in the patient	Ten repetitions for one set twice a day and progressing it up to 10 repetitions for three sets till week four
Walking exercises (six-minute walk test)	To enhance exercise tolerance	The therapist designed an incremental physical treatment regimen, starting with bedside sitting and progressing to bedside mobility exercises like walking for at least 10 minutes or to the patient’s tolerance. The therapist then gradually advanced the treatment based on the patient’s responsiveness and perceived exertion rate. The regimen was followed for four to seven days per week for 20–60 minutes until the heart rate reached 50–90% of its maximum
Orthotic management	To correct and further decline the progress of deformity	Milwaukee brace for six hours during the acute phase

Follow-up and outcomes

The therapist planned the physiotherapy program for two weeks, including four weekly sessions in the hospital inpatient setting. After two weeks, when the patient’s exercise tolerance capacity had improved, the patient was discharged with a well-explained home exercise routine and scheduled for a two-week follow-up. Table 4 shows the comparison between pre and post-rehabilitation assessments.

**Table 4:** The comparison between pre and post-rehabilitation assessments. NPRS: Numeric Pain Rating Scale; mMRC: Modified Medical Research Council; WHO-QOL: World Health Organization quality of life

Outcome measures	Pre-rehabilitation	Post-rehabilitation
NPRS	8/10	2/10
mMRC grade of dyspnea	Grade 2	Grade 1
WHO-QOL (physical functioning)	51/100	85/100
Six-minute walk test	180 m	300 m

## Discussion

In medical wards, empyema is a prevalent ailment. The accumulation of pus within the chest cavity leads to empyema. Pneumonia is the most prevalent cause of empyema. In the care of such individuals, physiotherapy or cardiorespiratory physiotherapy intervention is critical. An overall goal of a physiotherapy program is to use cutting-edge, economically viable therapeutic modalities, minimize patient dependence, enhance residual function, lower the likelihood of rehospitalization, and enhance the quality of life. Breathing exercises, particularly segmental breathing exercises, are a straightforward, low-cost, and simple-to-implement therapy [6].

Scoliosis treatment should focus on the functional and physiological difficulties it causes. Scoliosis has no acute symptoms or signals that necessitate surgery. It is critical to slow down the curve’s evolution and avoid the emergence of inappropriate postural habits. Early intervention, such as exercises and posture retraining, can help achieve this [7]. Sarkar et al. discovered that in restricted lung disorders such as empyema, breathing exercises such as segmental breathing are particularly helpful and can enhance lung expansion [8].

Controlling infection, draining the purulent fluid, and eradicating the sac to avoid chronicity and re-expansion of the damaged lung to restore function are the goals of pleural empyema therapy. The patient’s clinical status, the extent of the effusion, and imaging data determine the treatment. Antibiotics, recurring thoracentesis, tube thoracostomy, fibrinolytic agent therapy, decortication, and video-assisted thoracoscopic surgery (VATS) are all options for treating empyema. Some experts advocate antibiotics in conjunction with tube thoracostomy and intrapleural fibrinolytic medications as the best treatment for empyema. Others argue for early surgical intervention such as VATS [9].

## Conclusions

This study demonstrates that physiotherapy combined with medical care and drainage in inpatients with pleural effusion and scoliosis is associated with a shorter hospital stay and a better recovery. For patients with pleural effusion, adding physiotherapy based on mobilizations, deep breathing exercises, and incentive spirometry to the conventional treatment is possible and can improve pulmonary function and quality of life. In individuals with pleural effusion, there is evidence that physical therapy treatment reduces the severity of this illness with radiographic improvement. This study also demonstrates that orthotic management and physiotherapy can be used to treat scoliosis with clinically evident results.
